# Venous Thromboembolism and Cancer: A Comprehensive Review from Pathophysiology to Novel Treatment

**DOI:** 10.3390/biom12020259

**Published:** 2022-02-04

**Authors:** Mario Enrico Canonico, Ciro Santoro, Marisa Avvedimento, Giuseppe Giugliano, Giulia Elena Mandoli, Maria Prastaro, Anna Franzone, Raffaele Piccolo, Federica Ilardi, Matteo Cameli, Giovanni Esposito

**Affiliations:** 1 Department of Advanced Biomedical Sciences, Federico II University Hospital, 80131 Naples, Italy; marioenrico.canonico@unina.it (M.E.C.); marisa.avvedimento@unina.it (M.A.); giuseppe.giugliano@unina.it (G.G.); prastaro@unina.it (M.P.); anna.franzone@unina.it (A.F.); raffaele.piccolo@unina.it (R.P.); fedeilardi@gmail.com (F.I.); espogiov@unina.it (G.E.); 2 Division of Cardiology, Department of Medical Biotechnologies, University of Siena, 53100 Siena, Italy; giulia_elena@hotmail.it (G.E.M.); matteo.cameli@yahoo.com (M.C.); 3 Mediterranea Cardiocentro, 80122 Naples, Italy

**Keywords:** venous thromboembolism, cancer, anticoagulation, risk stratification, cardio-oncology

## Abstract

Acute thrombotic events can unveil occult cancer, as they are its first manifestation in about 20 to 30% of all cases. Malignancy interacts in an intricate way with the hemostatic system, promoting both thrombosis and bleeding. The main pathway involved in these reactions involves the activation of tumor-associated procoagulant factors, which eventually results in clot formation. The clinical manifestation of cancer-related thrombotic events mainly involves the venous side, and manifests in a broad spectrum of conditions, including unusual sites of venous thrombosis. The selection of patients who have a balanced risk–benefit profile for management of anticoagulation is complex, given individual patient goals and preferences, different prognosis of specific cancers, common comorbidities, potential drug–drug interactions, underweight states, and the competing risks of morbidity and mortality. Anticoagulant treatment in cancer settings is broadly debated, considering the potential application of direct oral anticoagulants in both thromboprophylaxis and secondary prevention, having demonstrated its efficacy and safety compared to conventional treatment. This review aims to provide a brief overview of the pathophysiology and management of cancer-related thrombosis, summarizing the results obtained in recent clinical trials.

## 1. Introduction

The intricate connection between malignancies and the hemostatic system has been known for a long time. Cancer cells override the coagulation pathway, to enhance their diffusion, by releasing procoagulant factors which eventually activate platelets and inflammatory cells, which in turn stimulate angiogenesis and clot formation. Indeed, thrombotic events could, as its first manifestation, unveil an occult malignancy [1]. Of interest, about 20 to 30% of all first venous thrombotic events are cancer related [2,3].

When a thrombotic event occurs, the presence of active malignancy is considered a determinant of unfavorable evolution, when compared to traditional counterparts. Therefore, in this setting, anticoagulation management should be prompt and aggressive. The decision on treatment duration should consider the higher recurrence risk present in an oncologic setting [4], as the presence of cancer itself is considered a “non-transient” risk factor for venous thromboembolism (VTE). On the other hand, cancer may increase the rate of bleeding events that account for higher mortality, with an incidence of 10% in solid tumors, and an even higher proportion in patients with hematologic malignancies [5]. Consequently, treatment of venous thromboembolism (VTE) in patients with cancer can be challenging, faced with serious complications including increased risk of bleeding, potential drug–drug interactions that accompany chemotherapy, underweight states, and the competing risks of morbidity and mortality.

## 2. Epidemiology and Risk Stratification

Several registries involved in the analysis of cancer and coagulation crosstalk provided precious information about the incidence of venous thrombosis in cancer settings [6,7,8,9,10]. In the Multiple Environmental and Genetic Assessment of risk factor for venous thrombosis (MEGA) study, more than 3000 patients who experienced DVT between 1999 and 2002 were included, and were matched with healthy controls. Among them, the risk of DVT was increased sevenfold by the presence of malignancies. Additionally, in patients with malignancies, the occurrence of DVT appeared to be high during the first year after cancer diagnosis, reaching its highest incidence within the first three months (adjusted OR, 53.5; 95% CI, 8.6–334.3) and decreasing as time progressed [11]. One possible explanation could lie in the natural course of the disease; cancer treatments (chemo- and hormonal therapy, need for blood transfusion, surgery, etc.), and their prothrombic effects, are concentrated within the first year after diagnosis, whilst subsequent cancer remission or death eventually reduce the occurrence of DVT.

Of interest, the incidence of cancer-related venous thrombosis showed a hyperbolic distribution over the last decades, with a 1.5% incidence rate in the late 1980s, and reaching 4.6% in the early 2000s [12]. This increase could be the consequence of different factors; firstly, improvements in diagnostic testing and the spreading acknowledgment of a solid link between cancer and venous thrombotic events; secondly, it could be the effect of multiple novel treatments with both direct and indirect iatrogenic effects, due to prolonged survival.

The risk of developing venous thrombotic events, in oncologic settings, depends on several cancer-related and patient-related factors (Table 1).

The type of malignancy seems to influence the risk of venous thrombosis, such that high-risk cancers (pancreas, brain, lung, ovarian, lymphoma, myeloma, kidney, stomach, and bone cancer) and low-risk cancers (breast, prostate, malignant melanoma, and testicular) can be identified [13]. Cancer features of biological aggressiveness, such as early metastatic diffusion and consequential dismal prognosis, correlate to incidence rates of venous thrombosis [14], as evidenced by Tim JF et al., who showed a positive association in incidence rates of venous thrombotic events plotted against one-year relative mortality for different types of cancer [15].

In oncologic patients, several conditions not associated with cancer, defined as patient-related risk factors, might also increase the risk of thrombotic events. Among them, age and history of previous venous thrombosis are the most recognized. Additionally, as demonstrated in a retrospective analysis of 68,142 colorectal cancer patients, the concomitant presence of chronic comorbidities (such as pulmonary and renal disease, infection, and anemia) increases the risk of venous thrombosis by a factor of two within one year after diagnosis [16]. Other common risk factors for venous thrombosis in non-cancer populations, which may recur in this context, such as prolonged immobility after a surgical treatment, placement of central venous catheters (CVC), or prothrombotic mutations, should also be a concern when assessing thrombotic risk in cancer patients. [11,17].

## 3. Biological Mechanisms of Cancer-Related Thrombosis

**Figure 1:**
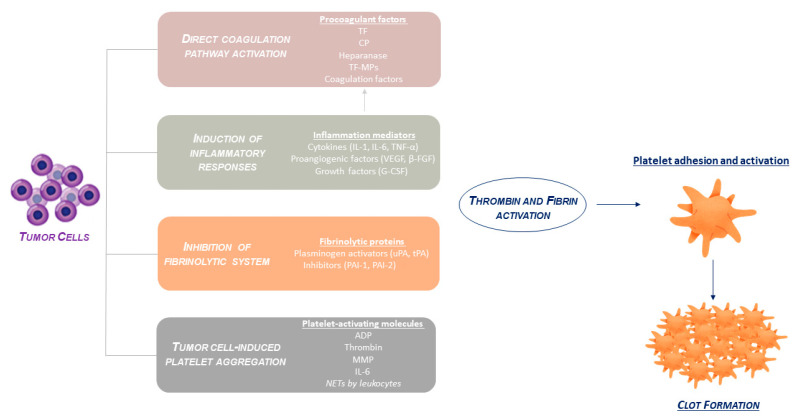
Crosstalk between malignant cells and the hemostatic system.

Malignancy interacts in an intricate way with the hemostatic system, enabling both thrombosis and hemorrhage. Clinical factors, combined with disease-specific features and biological procoagulant mechanisms, contribute to the definition of overall thrombotic risk in patients with cancer. The pathogenesis of cancer-induced VTE is multifactorial and involves several overlapping pathways, including direct coagulation pathway activation, the induction of inflammatory responses, the inhibition of fibrinolytic activity, and tumor cell-induced platelet aggregation (Figure 1).

### 3.1. Direct Coagulation Pathway Activation

Tumor cells release a variety of procoagulant substances, such as tissue factor (TF), TF-positive tumor-derived microparticles (MPs), cancer procoagulant (CP) factor, and heparinase, resulting in a hypercoagulable state [18]. The up-regulation of TF, a primary initiator of the coagulation cascade, has been suggested as the main mechanism through which cancer induces fibrin formation. It forms a complex with activated factor VII to trigger blood coagulation by proteolytic activation of factors IX and X. Procoagulant TF can be vacuolized and released in tumor microparticles rich in TF, which disseminate into the body and cause both systemic and localized thrombotic events. Mice injected with TF-enriched microparticles showed a higher incidence of disseminated intravascular coagulation (DIC)-like syndrome, thus reinforcing this pathophysiological hypothesis [19]. Different cancer histotypes constitutively overexpress TF, higher expression levels of which seem to be associated with a more aggressive pattern of tumor growth and vascularity.

- Moreover, CP, which is highly expressed by proliferating blast cells and some solid tumors, can directly activate Factor X, shifting the hemostatic balance towards a prothrombotic condition [20,21].
- MPs represent an emerging mechanism of cancer-promoted clotting activation. MPs are fragments shed from the plasma membrane of numerous cell types—including tumors, blood cells, and endothelial cells—on exposure to stress conditions, and act as mediators of cell–cell communication [22]. Furthermore, MPs can also play a role in cancer progression, due to their ability to positively influence angiogenesis [23].
- Angiogenesis plays a significant role in tumor progression, and, to date, evidence from several studies supports the use of low molecular weight heparin (LMWH) as a therapeutic tool in cancer patients, by utilizing the anti-metastatic properties of LMWH [24]. This effect seems to be linked to different synergistic mechanisms, among which are the prevention of cancer-induced hypercoagulation, cancer cell proliferation, apoptosis, and angiogenesis [25]. Specifically, LMWH expresses anti-angiogenic activity via binding and inhibiting angiogenic growth factors, such as Vascular Endothelial Growth factor (VEGF), and interferes with fibrin formation, resulting in inhibition of cancer metastasis [26].

### 3.2. Induction of Inflammatory Responses

The production of proinflammatory cytokines (interleukin 1b (IL-1b), IL-6, IL-1β, tumor necrosis factor a (TNF-a), and lipopolysaccharides) and proangiogenic factors (mainly vascular endothelial growth factor (VEGF) and fibroblast growth factor (FGF)) by malignant cells further contributes to pro-coagulant status by inducing cell adhesion and vascular cell activation [27]. It has been extensively demonstrated that interleukin levels are significantly elevated in cancer patients compared to healthy controls, especially in those with VTE. The inflammatory response, through increased release of TF, von Willebrand factor, plasminogen activator inhibitor (PAI-1), and VEGF induced by endothelial cells, together with a down-regulation of thrombin activity and the protein C system, can lead to a procoagulant phenotype in patients with cancer.

Tumor cells can also activate leukocytes by direct cell–cell adhesion. Neutrophils and monocytes/macrophages activate release procoagulant enzymes and expose, on their surface, high levels of TF and adhesion molecules for platelets and endothelial cells, thus leading to fibrin formation and deposition. Furthermore, recent evidence suggests that inflammatory responses to cancer can also result in the formation of neutrophil extracellular traps (NETs), which are scaffolds made of externalized DNA, histones, and proteases. NETs have a strong pro-thrombotic effect, providing a stimulus for platelet adhesion and aggregation; conversely, activated platelets can promote their formation and release.

### 3.3. Inhibition of Fibrinolytic System

In addition to the induction of fibrin clots, cancer can also result in an anticoagulant effect by interacting with the fibrinolytic system. Indeed, cancer tissue can express fibrinolytic proteins, such as the plasminogen activators (urokinase-type plasminogen activator (uPA) and tissue-type plasminogen activator (tPA)), their inhibitors (PAI-1 and -2), and their receptors. PAI-1 is the major inhibitor of plasminogen activation by tPA, and therefore is an inhibitor of fibrin clot degradation [28]. Elevated levels of PAI-1 antigen and activity have been found in patients with cancer, which was combined to VTE susceptibility [29].

### 3.4. Tumor Cell-Induced Platelet Aggregation

There is growing evidence that platelet function plays an important role in promoting the hypercoagulable state of patients with cancer [30]. Tumor cells activate platelets through direct cancer cell–platelet adhesion, and/or by tumor secretion of platelet-activating molecules (i.e., ADP, thrombin, matrix metalloproteinases, IL-6) which lead to platelet adhesion/aggregation [31]. Among adhesion mechanisms, selectins expressed on platelets, leukocytes, and endothelium tissue can bind tumor cells to form aggregates. Specifically, P-selectin, expressed on the surface of activated platelets, binds to many types of human cancer cells, and this interaction can also promote tumor growth and metastasis [32]. Activated platelets can mediate the onset of hypercoagulability in cancer patients by direct clotting activation and thrombus formation, or by interacting with other blood cells. As reported, platelets stimulate the release of NETs via leukocytes, and their interaction with endothelial cells is relevant in platelet-mediated cancer-associated VTE.

## 4. Clinical Presentation

Cancer-related thrombotic events usually occur on the venous side, and manifest clinically in a broad spectrum of conditions, also addressed as Trousseau’s syndrome, that includes venous and arterial thrombosis, non-bacterial thrombotic endocarditis (NBTE), thrombotic microangiopathy (TMA), and veno-occlusive disease (VOD).

**Figure 2:**
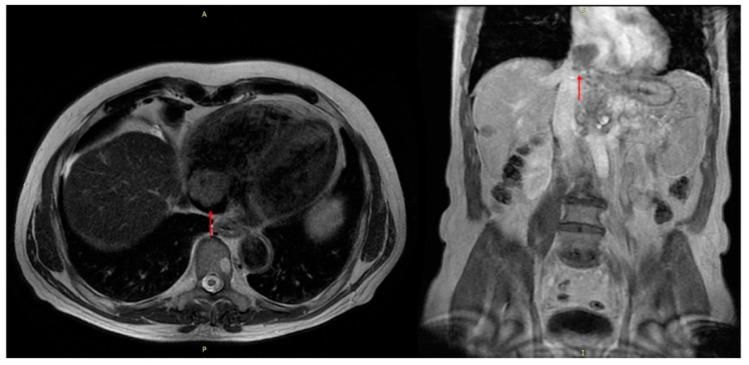
CT scan in patients with hepatocarcinoma. Red arrow: extensive thrombosis, originating in the hepatic veins (not visible in these scans), invading the right atrium through the inferior vena cava.

Venous thrombosis usually effects lower limbs, and clinical presentation in cancer patients may not differ from those patients without cancer. However, cancer related DVT may manifests in several sites (iliocaval, portal or extrahepatic, mesenteric, upper limb veins, migratory superficial thrombophlebitis) whose singularity should draw attention to possible unknown malignancies [33]. (Figure 2). Specifically, the presence of indwelling vascular access devices, such as a peripheral inserted central catheter (PICC) or CVC, subject to periodical anticancer infusions, could represent a source of upper limb and/or superior vena cava thrombosis, due to thrombogenic catheter material, larger catheter diameter, and greater number of lumens.

## 5. Treatment

### 5.1. Thromboprophylaxis

The use of thromboprophylaxis for primary prevention of VTE is an important clinical and research issue. Extended treatment with low molecular weight heparin (LMWH) or direct oral anticoagulants (DOACs) after discharge of cancer patients decreased VTE events, at the cost of increased bleeding [34]. Several scores have been developed to assess the risk of cancer-related VTE. A systematic review and meta-analysis examined the performance of the Khorana score in predicting VTE in over 34,000 ambulatory patients with various types of cancer. In the first six-month follow-up, the risk of VTE in patients with a high-risk Khorana score was 11.0%, which was significantly higher than in those with a low-risk (5.0%) or intermediate-risk (6.6%) score. These results indicate that the Khorana score may help clinicians in selecting patients at high risk of VTE for thromboprophylaxis; specifically, those with a Khorana score ≥ 2 and low bleeding risk [35].

Moreover, Khorana et al. developed a risk model based on five variables assessed using a cohort study of 2701 cancer patients who were beginning chemotherapy. Patients with a risk score higher than three (i.e., high risk group) before starting the treatment had a 6.7 to 7.1% risk of developing venous thrombosis [36]. Furthermore, improvements in the prediction of thrombotic events have been achieved by adding bio-humoral markers (i.e., P-selectin > 53.1 ng/mL and D-dimer levels > 1.44 mg/mL) to the Khorana’s risk model. This expanded model showed a sensitivity of 96% when patients receive a point score of one, thus reasonably excluding thromboprophylaxis, and a specificity of 98% for those at a higher cut off point (score ≥ 5) who may benefit from thromboprophylaxis [33]. A limitation of this expanded score is the scarce availability of those bio-humoral markers in daily routine.

In each phase of treatment, from thromboprophylaxis to secondary prevention, adverse bleeding events represent a challenge in clinical practice. For the International Society on Thrombosis and Hemostasis, major bleeding is defined as bleeding which caused a decrease in hemoglobin levels of 2 grams per deciliter or more, led to the transfusion of two or more units of packed red cells, occurred in a critical site (intracranial, intra-spinal, intraocular, pericardial, or retroperitoneal bleeding), or contributed to death [37]. Clinically relevant non-major bleeding (CRNMB) represents a bleeding episode that did not meet the criteria for major bleeding, but was associated with medical intervention (contact with a physician, interruption or discontinuation of the assigned treatment, or discomfort in or impairment of daily life activities [37].

To date, the AVERT and CASSINI trials represent the only trials investigating DOAC effects in cancer patients who may benefit from thromboprophylaxis. These trials investigated the efficacy and safety of six months of treatment with apixaban (2.5 mg twice daily) and rivaroxaban (10 mg once daily), versus placebo, in the prevention of VTE in high-risk ambulatory patients assessed by the Khorana score [37,38]. In the AVERT trial, VTE occurred in 4.2% of the apixaban arm and in 10.2% of the placebo arm (Hazard Ratio (HR) 0.41, 95% CI, 0.26–0.65, *p* < 0.001), while, in an intention-to-treat analysis, major bleeding occurred in 3.5% and 1.8% of the apixaban and placebo groups, respectively (HR 2.00, 95% CI, 1.01–3.95, *p* = 0.046) [33]. In the CASSINI study, the primary endpoint (a composite of DVT, PE, and death from VTE) showed no difference between the rivaroxaban group (6.0%) and the placebo group (8.8%), (HR 0.66, 95% CI, 0.40–1.09, *p* = 0.10) as well as no difference in the rates of major bleeding, which occurred in 2.0% of the rivaroxaban arm and 1.0% of the placebo arm (HR 1.96, 95% CI, 0.59–6.49, *p* = 0.26) [38]. In a cumulative analysis comparing the results of these two trials, Agnelli showed a significant benefit of direct oral anticoagulants for the prevention of venous thromboembolism, with a low incidence of major bleeding [39].

### 5.2. Acute Treatment of VTE and Secondary Prevention

The selection of patients who have a balanced risk–benefit profile for initiation of anticoagulation is complex, given individual patient goals and preferences, changing prognoses of specific cancers, common comorbidities, potential drug–drug interactions, underweight states, and the competing risks of morbidity and mortality [40].

The choice of which anticoagulation protocol to apply in the presence of active cancer should account for potential drug–drug interactions, interference in intestinal tablet absorption, and the possibility of prompt counteraction, in case of adverse events. The use of parental LMWH, instead of a Vitamin K antagonist (VKA) or DOAC, can help to reduce interactions with some anticancer drugs and avoid pharmacokinetic interference due to chemotherapy-induced nausea and vomiting, as well as increasing the possibility of closer monitoring [41]. Drug–drug interactions between DOACs and anticancer agents include cytochrome P450 3A4 (CYP 3A4) and P-glycoprotein (P-gp). These interactions involve several anticancer drug classes, such as antimitotic microtubule inhibitors, tyrosine kinase inhibitors, cyclosporine, topoisomerase inhibitors, anthracyclines, and hormone agents [39]. Renal function impairment, including acute and chronic kidney disease, is not uncommon in cancer patients, and affects both LMWH and DOAC pharmacokinetics. Individual agents have differing renal clearance rates (dabigatran, 80%; edoxaban, 50%; rivaroxaban, 33%; and apixaban, 27%). Appropriate renal dose adjustment is critical for patients with renal dysfunction and concomitant DOAC administration; however, LMWH doses should be adjusted as well [41]. Thrombocytopenia often affects patients with myeloproliferative malignancy and metastasis of bone marrow. In this setting, anticoagulation should be strictly monitored when platelet counts are below 50.000 uL [42]. Finally, heparin-induced thrombocytopenia represents a common consequence, which can be bypassed by using fondaparinux.

In the pre-DOAC era, clinical trials in this population compared LMWH monotherapy to LMWH-bridged warfarin. In the CLOT trial, after initial treatment with LMWH, 672 cancer patients were randomly assigned to dalteparin treatment (200 IU/kg in the first month, followed by 125 IU/kg) or VKA (International Normalized Ratio (INR) target 2.5) for six months. The study revealed the superior efficacy of dalteparin compared to vitamin K antagonists, with no increase in major bleeding events (6 versus 4%, *p* = 0.27) and a substantial reduction in recurrent VTE rates observed in the dalteparin-treated group (9 versus 17%; HR 0.48, 95% CI, 0.30–0.77, *p* = 0.002) [43].

The CATCH study, which compared tinzaparin to warfarin in open label randomization, showed that recurrent VTE occurred (in 6 months) in 7.2% of the tinzaparinarm vs. 10.5% of the warfarin arm (0.65 HR, 95% CI, 0.41–1.03; *p* = 0.07). There were no differences in major bleeding rates (HR 0.89, 95% CI, 0.40–1.99; *p* = 0.77) or overall mortality (*p* = 0.54). A significant reduction in CRNMB was observed with tinzaparin (*p* = 0.004) [44].

Recently, the use of DOACs has increased in treatment of VTE, prompting the use of DOACs in cancer patients with VTE (Table 2). In the Hokusai VTE Cancer study, 1050 cancer patients with VTE, after 5 days of LMWH treatment, were randomized in an open-label fashion and assigned to either an edoxaban arm (60 or 30 mg in patients meeting criteria for dose reduction) or the continuing dalteparin arm. During the 12 month follow-up period, edoxaban was non-inferior to dalteparin with respect to composite recurrent VTE and major bleeding rates, which were 12.8% vs. 13.5%, (HR 0.97, 95% CI, 0.70–1.36, *p* = 0.006). Recurrent VTE was reduced by edoxaban compared with dalteparin (7.9% vs. 11.3%), but major bleeding rates increased (6.9% vs. 4.0%), driven by higher bleeding rates in patients with gastrointestinal cancers. Mortality rates at 12 months were 39.5% (edoxaban arm) and 36.6% (dalteparin arm). This trial shows that edoxaban could be an attractive alternative to subcutaneous dalteparin for the treatment of the majority of patients with cancer-associated VTE; however, caution should be advised in subjects with gastrointestinal malignancy due to an increased risk of major bleeding [45].

In the Select-D Pilot trial, an open-label RCT of 406 patients with cancer (mainly colorectal, lung, and breast cancer) and VTE, patients were randomly assigned in a 1:1 ratio to a rivaroxaban arm (15 mg twice daily for 3 weeks followed by 20 mg daily) or dalteparin arm (200 UI/kg once daily for 30 days, followed by 150/UI daily) for 6 months. Rivaroxaban reduced the risk of recurrent VTE (4% vs. 11%, HR 0.43, 95% CI 0.19–0.99), but increased the risk of CRNMB (13% vs. 4%, HR 3.76, 95% CI 1.63–8.69) compared to dalteparin. Overall 6-month survival rates were comparable (75% and 70% for rivaroxaban and dalteparin, respectively) [46].

In the AMPLIFY trial, apixaban (10 mg twice daily for 7 days, followed by 5 mg twice daily) was compared to enoxaparin (1 mg/kg twice daily for at least 5 days) followed by warfarin (INR for 2–3) for the treatment of acute venous thromboembolism. Of the 5395 patients that were randomized, about 10% had active cancer or history of cancer. Among patients with active cancer, recurrent VTE occurred in 3.7% and 6.4% of patients in the apixaban and enoxaparin/warfarin groups, respectively (relative risk (RR) 0.56, 95% CI 0.13–2.37); major bleeding occurred in 2.3% and 5.0% of evaluable patients, respectively (RR 0.45, 95% CI 0.08–2.46). The result of the subgroup analysis of cancer patients showed that apixaban may be an interesting option for this category [51].

In the ADAM VTE trial, patients with cancer-associated VTE were randomly assigned to receive either apixaban (10 mg twice daily for seven days, followed by 5 mg twice daily) or subcutaneous dalteparin (200 IU/kg once daily for the first month, followed by 150 IU/kg for months 2 through 6) for six months. Recurrent VTE occurred in 0.7% and 6.3% of patients in the apixaban and dalteparin groups, respectively (HR 0.09, 95% CI 0.013–0.780, *p* = 0.028). Major bleeding was observed in 0% of the apixaban arm vs. 1.4% of the dalteparin arm (*p* = 0.138). Apixaban was associated with low major bleeding and VTE recurrence rates in cancer patients [47]. 

The Caravaggio trial was a multinational, randomized, investigator-initiated, open-label, noninferiority trial including 1155 patients with cancer and symptomatic or incidental proximal DVT or PE. Patients were randomized to receive apixaban (10 mg twice daily for the first 7 days, followed by 5 mg twice daily) or dalteparin (200 UI/kg once daily for 30 days, followed by 150/UI daily) for 6 months. Recurrent VTE occurred in 5.6% of the apixaban group and 7.9% of the dalteparin group (HR, 0.63; 95% CI, 0.37–1.07; *p* < 0.001). Major bleeding occurred in 3.8% of the apixaban group and 4.0% of the dalteparin group (HR, 0.82; 95% CI, 0.40 to 1.69; *p* = 0.60). Apixaban was shown to be noninferior to subcutaneous dalteparin for the treatment of cancer-associated venous thromboembolism, without an increased risk of major bleeding [48].

More recently, the CASTA-DIVA study, a randomized open-label non-inferiority trial, included 158 patients with active cancer who had proximal VTE; patients were assigned to receive rivaroxaban (15 mg twice daily for 21 days, followed by 20 mg once daily) or dalteparin (200 UI/kg once daily for 30 days, followed by 150/UI daily) for 3 months. Recurrent VTE occurred in four patients in the rivaroxaban arm and in six patients in the dalteparin arm (HR, 0.75; 95% CI, 0.21–2.66; *p* = 0.13 for noninferiority). Regarding safety profiles, major bleeding or CRNMB occurred in nine and eight patients in the rivaroxaban and dalteparin groups, respectively (HR, 1.27; 95% CI, 0.49–3.26) [49].

In addition, the preliminary results of the CANVAS pragmatic randomized trial showed a non-inferiority of recurrent VTE treatment efficacy in 811 randomized cancer patients treated with DOACs, vs. LMWH. After 6 months, the VTE rates were similar between groups (DOACs, 6.4% vs. LMWH, 7.8%) (HR −1.3; 95% CI, −4.4–1.7), with no differences in major bleeding rates (DOACs, 5.4% vs. LMWH, 4.4%) (HR −1.0 95% CI, −1.5–3.6) [50].

Moreover, two new trials (COSIMO and CONKO-011) investigated patient-reported treatment satisfaction with rivaroxaban, as compared to LMWH, for cancer patients with VTE. CONKO-011 randomized 247 patients into the two arms. Anti-Clot Treatment Scale (ACTS) Burdens scores, after 4 weeks, were 52.8 versus 51.2 for rivaroxaban vs. LMWH (*p* = 0.006), showing improved patient-reported treatment satisfaction with rivaroxaban [52]. COSIMO was a non-interventional study with 505 subjects enrolled. The results showed patients’ treatment satisfaction following a planned switch from traditional therapy (LMWH/VKA/fondaparinux) to rivaroxaban in VTE cancer patients. Mean ACTS Burdens scores were higher until 6 months after the beginning of rivaroxaban therapy, compared to baseline (55.6 vs. 51.8, respectively; *p* < 0.0001 at 4 weeks), confirming improvement of patient-reported anticoagulation burden [53].

The International Society on Thrombosis and Hemostasis (ISTH) and National Comprehensive Cancer Network (NCCN) 2018 guidelines recommend LMWH as the standard of care in treating cancer-associated VTE, with fondaparinux and unfractionated heparin as alternative treatment options, compared to oral anticoagulant treatment with warfarin [54].

The 2019 ASCO Clinical Practice Guideline Update underscored the importance of risk stratification for VTE risk, as well as the importance of effective treatment to reduce the risk of VTE recurrence and mortality [55].

It stands to reason that the occurrence of cancer heavily affects all three phases of the anticoagulation management of DVT; initial management (consisting of the first 5 to 21 days), primary treatment (the 3 to 6 months following initial management), and secondary prevention (the duration of which depend on VTE recurrency), as classified by the recent guidelines for management of venous thromboembolism [55].

Anticoagulant treatment DOACs provide a reasonable alternative to LMWH in the treatment of VTE in cancer patients; however, particular care should be taken to monitor bleeding events, especially those that are gastrointestinal or urinary [56].

**Figure 3:**
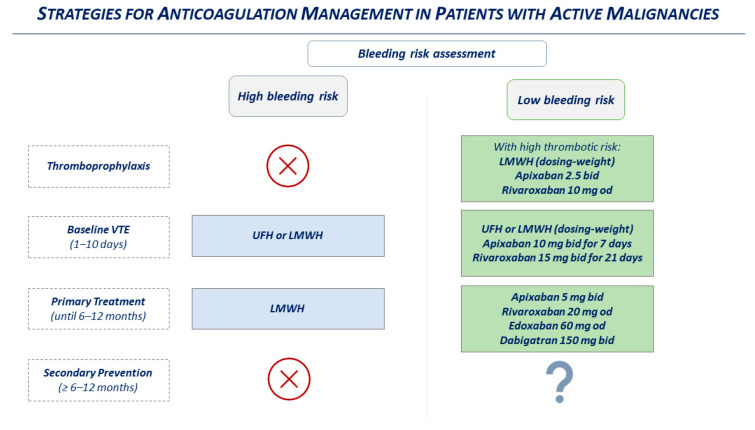
Strategies for anticoagulation management in patients with active malignancies. UFH, unfractionated heparin; LMWH, low molecular weight heparin; Od, once daily; Bid, bis in die.

An unsolved question that remains is the optimal duration of anticoagulant therapy in cancer patients. After the acute phase of VTE (3–6 months), it is reasonable to extend the anticoagulant regimen for high-risk patients until 12 months or longer. Indeed, the trials investigating the management of secondary prevention after venous thrombotic events are the RE-MEDY, RE-SONATE, EINSTEIN-EXT, EINSTEN-CHOISE, and Amplify-EXT trials, which evaluate the efficacy of DOACs in preventing recurrent venous thrombosis after 6 months. In the RE-MEDY and RE-SONATE trials, dabigatran vs. warfarin or placebo, respectively, showed a reduction of recurrent VTE with similar rates of major bleeding [57]. In the EISTEIN-EXT and EINSTEN-CHOISE trials, rivaroxaban reduced the risk of recurrent VTE without a difference in major bleeding rates compared to placebo or aspirin, respectively; in the Amplify-EXT trial, apixaban showed the same results versus placebo [57]. However, active malignancies were considered an exclusion criterion in these trials; therefore, these results cannot be extrapolated to the oncologic population. A dedicated study on this issue should be conducted to acquire these protocols in clinical practice (Figure 3).

## 6. Prognosis

The concomitant presence of cancer and a thromboembolic event reduces survival rates fivefold, when compared to a thrombotic event alone, at 1 year [13]. On the other hand, the survival rate of patients with cancer and venous thrombosis is more than halved at 1 year if compared to cancer populations without venous thrombosis, after matching for age, gender, type of cancer, and year of diagnosis [58]. Indeed, venous thrombosis is a significant prognosticator at 1 year for all cancer types, in a large cohort study of more than 230 thousand cancer patients, with and without thrombotic events [21]. Even though the specific weight of thrombotic events alone on mortality rates in cancer populations is difficult to estimate, thrombotic events are the second most common cause of death in patients with cancer, after cancer progression itself [59].

## 7. Conclusions

Cancer-related venous thrombotic events are a common clinical manifestation during the course of disease. The pathogenesis of cancer-induced VTE is multifactorial, and involves several pathways, including direct coagulation pathway activation, the induction of inflammatory responses, the inhibition of fibrinolytic activity, and tumor cell-induced platelet aggregation. The right choice of treatment, between anticoagulation strategy, thrombo-hemorrhagic risk management, and patients’ comorbidities, represents a challenge for physicians. An accurate risk stratification, to select patients at higher risk for thrombotic events who would benefit from thromboprophylaxis, should be encouraged. Early identification and treatment of this complication is particularly relevant in the onco-hematologic setting, given the substantial impact of venous thrombotic events on morbidity and mortality. The “one fits all” choice of LMWH is giving way to DOAC strategy, in specific settings. Indeed, despite some evidence of increased risk of major bleeding, DOACs are an attractive alternative to LMWH in the treatment of VTE in cancer patients, especially those without drug interactions, and those with significantly impaired renal function. The recent Sars-CoV2 pandemic has drawn attention to the management of venous and arterial thromboembolism, an issue that is even more compelling in patients affected by both cancer and COVID 19, who require intensive care [60]. The large amount of multiple connections between the thrombotic pathway and cancer growth needs to be furtherly elucidated, today more than ever, in order to provide accurate prognostic scores and data-driven decisions for this vulnerable population.

## Figures and Tables

**Table 1:** Risk factors for venous thrombosis.

**Cancer-Related**	**Patient-Related**
Type of cancer - High risk: pancreas, brain, lung, ovarian, lymphoma, myeloma, kidney, stomach and bone cancer; - Low risk: breast, prostate, melanoma, testicular cancer. Stage of cancer - Advanced stage and initial period after diagnosis. Treatment - Chemo- and hormonal therapy; - Anti-angiogenic therapy; - Erythropoiesis stimulating agents; - Blood transfusions.	Older age. Prolonged immobility. Prior history of venous thrombosis. Black ethnicity. Prothrombotic mutation. Indwelling catheter. Comorbidities (≥3): - Arterial thromboembolism; - Pulmonary disease; - Renal disease; - Infection; - Anemia.

**Table 2:** Studies investigating the role of DOACs in VTE cancer patients.

Study	Type of Study	Phase	Cancer Patients Enrolled	Type of Cancer Excluded	Drugs	Follow-Up	Main results	
							Rate of VTE Bleeding	
AVERT [37]	Randomized double-blind vs. Placebo	Thromboprophylaxis	574	Basal cell and squamous cell carcinoma and acute leukemia.	Apixaban vs. Placebo	6 months	Apixaban vs. Placebo: 4.2% vs. 10.2%, HR 0.41 (0.26–0.65)	Apixaban vs. Placebo: 3.5% vs. 1.8% HR 2.00 (1.01–3.95)
CASSINI [38]	Randomized double-blind vs. Placebo	Thromboprophylaxis	1080	Brain (local or metastases) and hematologic malignances (except lymphoma).	Rivaroxaban vs. Placebo	6 months	Rivaroxaban vs. Placebo: 6.0% vs. 8.8% HR 0.66 (0.40–1.09)	Rivaroxaban vs. Placebo: 2.0% vs. 1.0% HR 1.96 (0.59–6.49)
Hokusai-VTE cancer [45]	Randomized open-label vs. Dalteparin	Primary treatment	1050	Basal-cell or squamous-cell carcinoma of the skin.	Edoxaban vs. Dalteparin	12 months	Edoxaban vs. Dalteparin: 7.9% vs. 11.3% HR 0.71 (0.48–1.06)	Edoxaban vs. Dalteparin: 6.9% vs. 4.0% HR 1.77 (1.03–3.04)
SELECT-D [46]	Randomized open-label vs. Dalteparin	Primary treatment	406	Basal-cell or squamous-cell carcinoma of the skin.	Rivaroxaban vs. Dalteparin	6 months	Rivaroxaban vs. Dalteparin: 4% vs. 11% HR 0.43 (0.19–0.99)	Rivaroxaban vs. Dalteparin: 6% vs. 4% HR 1.83 (0.68–4.96)
ADAM VTE [47]	Randomized open-label vs. Dalteparin	Primary treatment	300	None.	Apixaban vs. Dalteparin	6 months	Apixaban vs. Dalteparin: 6% vs. 6% HR 0.93 (0.43–2.02)	Apixaban vs. Dalteparin: 0% vs. 1.4%, HR: NA
Caravaggio [48]	Randomized open-label vs. Dalteparin	Primary treatment	1155	Basal-cell or squamous-cell carcinoma of the skin, primary brain tumor or known intracerebral metastases, and acute leukemia.	Apixaban vs. Dalteparin	6 months	Apixaban vs. Dalteparin: 5.6% vs. 7.9% HR 0.63 (0.37–1.07)	Apixaban vs. Dalteparin: 3.8% vs. 4.0%, HR: 0.82 (0.40–1.69)
CASTA-DIVA [49]	Randomized open-label vs. Dalteparin	Primary treatment	158	None.	Rivaroxaban vs. Dalteparin	6 months	Rivaroxaban vs. Dalteparin: 6.4% vs. 10.1% HR 0.75 (0.21–2.66)	Rivaroxaban vs. Dalteparin: 12.2% vs. 9.8% HR 1.27 (0.49–3.26)
CANVAS [50]	Randomized open-label vs. LMWH	Primary treatment	811	Acute leukemia.	DOACs vs. LWMH	6 months	DOACs vs. LWMH: 6.4% vs. 7.8% HR −1.3 (−4.4–1.7)	DOACs vs. LWMH: 5.4% vs. 4.4% HR −1.0 (−1.5–3.6)

## Data Availability

Not applicable.

## References

[1] Carrier M., Le Gal G., Wells P.S., Fergusson D., Ramsay T., Rodger M.A. (2008). Systematic review: The Trousseau syndrome revisited: Should we screen extensively for cancer in patients with venous thromboembolism?. Ann. Intern. Med..

[2] Spencer F.A., Lessard D., Emery C., Reed G., Goldberg R.J. (2007). Venous thromboembolism in the outpatient setting. Arch. Intern. Med..

[3] White R.H., Zhou H., Murin S., Harvey D. (2005). Effect of ethnicity and gender on the incidence of venous thromboembolism in a diverse population in California in 1996. Thromb. Haemost..

[4] Ortel T.L., Neumann I., Ageno W., Beyth R., Clark N.P., Cuker A., Hutten B.A., Jaff M.R., Manja V., Schulman S. (2020). American Society of Hematology 2020 guidelines for management of venous thromboembolism: Treatment of deep vein thrombosis and pulmonary embolism. Blood Adv..

[5] Reeves B.N., Key N.S. (2012). Acquired hemophilia in malignancy. Thromb. Res..

[6] Braekkan S.K., Borch K.H., Mathiesen E.B., Njølstad I., Wilsgaard T., Hansen J.B. (2010). Body height and risk of venous thromboembolism: The Tromsø Study. Am. J. Epidemiol..

[7] Gussoni G., Frasson S., La Regina M., Di Micco P., Monreal M., RIETE Investigators (2013). Three-month mortality rate and clinical predictors in patients with venous thromboembolism and cancer. Findings from the RIETE registry. Thromb. Res..

[8] Heit J.A., O’Fallon W.M., Petterson T.M., Lohse C.M., Silverstein M.D., Mohr D.N., Melton L.J. (2002). Relative impact of risk factors for deep vein thrombosis and pulmonary embolism: A population-based study. Arch. Intern. Med..

[9] Imberti D., Agnelli G., Ageno W., Moia M., Palareti G., Pistelli R., Rossi R., Verso M., MASTER Investigators (2008). Clinical characteristics and management of cancer-associated acute venous thromboembolism: Findings from the MASTER Registry. Haematologica.

[10] Ohashi Y., Ikeda M., Kunitoh H., Sasako M., Okusaka T., Mukai H., Fujiwara K., Nakamura M., Oba M.S., Kimura T. (2020). Venous thromboembolism in cancer patients: Report of baseline data from the multicentre, prospective Cancer-VTE Registry. Jpn. J. Clin. Oncol..

[11] Blom J.W., Doggen C.J., Osanto S., Rosendaal F.R. (2005). Malignancies, prothrombotic mutations, and the risk of venous thrombosis. JAMA.

[12] Khorana A.A., Francis C.W., Culakova E., Kuderer N.M., Lyman G.H. (2007). Frequency, risk factors, and trends for venous thromboembolism among hospitalized cancer patients. Cancer.

[13] Cronin-Fenton D.P., Søndergaard F., Pedersen L.A., Fryzek J.P., Cetin K., Acquavella J., Baron J.A., Sørensen H.T. (2010). Hospitalisation for venous thromboembolism in cancer patients and the general population: A population-based cohort study in Denmark, 1997–2006. Br. J. Cancer.

[14] Wun T., White R.H. (2009). Epidemiology of cancer-related venous thromboembolism. Best Pract. Res. Clin. Haematol..

[15] Timp J.F., Braekkan S.K., Versteeg H.H., Cannegieter S.C. (2013). Epidemiology of cancer-associated venous thrombosis. Blood.

[16] Alcalay A., Wun T., Khatri V., Chew H.K., Harvey D., Zhou H., White R.H. (2006). Venous thromboembolism in patients with colorectal cancer: Incidence and effect on survival. J. Clin. Oncol..

[17] Dentali F., Gianni M., Agnelli G., Ageno W. (2008). Association between inherited thrombophilic abnormalities and central venous catheter thrombosis in patients with cancer: A metaanalysis. J. Thromb. Haemost..

[18] Santoro C., Capone V., Canonico M.E., Gargiulo G., Esposito R., Sanna G.D., Parodi G., Esposito G. (2021). Single, Dual, and Triple Antithrombotic Therapy in Cancer Patients with Coronary Artery Disease: Searching for Evidence and Personalized Approaches. Semin. Thromb. Hemost..

[19] Falanga A., Tartari C.J., Marchetti M. (2012). Microparticles in tumor progression. Thromb. Res..

[20] Kozwich D.L., Kramer L.C., Mielicki W.P., Fotopoulos S.S., Gordon S.G. (1994). Application of cancer procoagulant as an early detection tumor marker. Cancer.

[21] Molnar S., Guglielmone H., Lavarda M., Rizzi M.L., Jarchum G. (2007). Procoagulant factors in patients with cancer. Hematology.

[22] Zwicker J.I., Trenor C.C., Furie B.C., Furie B. (2011). Tissue factor-bearing microparticles and thrombus formation. Arterioscler. Thromb. Vasc. Biol..

[23] Martinez M.C., Andriantsitohaina R. (2011). Microparticles in angiogenesis: Therapeutic potential. Circ. Res..

[24] Chao B.H., Lepeak L., Leal T., Robins H.I. (2011). Clinical use of the low-molecular-weight heparins in cancer patients: Focus on the improved patient outcomes. Thrombosis.

[25] Norrby K. (2006). Low-molecular-weight heparins and angiogenesis. APMIS.

[26] Sumanasekera W., Nethery W., Tran L., Pillai G. (2018). Low Molecular Weight Heparin as a Therapeutic Tool for Cancer; Special Emphasis on Breast Cancer. Biomed. J. Sci. Tech. Res..

[27] Falanga A., Panova-Noeva M., Russo L. (2009). Procoagulantmechanisms in tumour cells. Best Pract. Res. Clin. Haematol..

[28] Binder B.R., Christ G., Gruber F., Grubic N., Hufnagl P., Krebs M., Mihaly J., Prager G.W. (2002). Plasminogen activator inhibitor 1: Physiological and pathophysiological roles. News Physiol. Sci..

[29] Casslén B., Bossmar T., Lecander I., Astedt B. (1994). Plasminogen activators and plasminogen activator inhibitors in blood and tumour fluids of patients with ovarian cancer. Eur. J. Cancer.

[30] Connolly G.C., Phipps R.P., Francis C.W. (2014). Platelets and cancer-associated thrombosis. Semin. Oncol..

[31] Lee E.C., Cameron S.J. (2017). Cancer and Thrombotic Risk: The Platelet Paradigm. Front. Cardiovasc. Med..

[32] Chen M., Geng J.G. (2006). P-selectin mediates adhesion of leukocytes, platelets, and cancer cells in inflammation, thrombosis, and cancer growth and metastasis. Arch. Immunol. Ther. Exp..

[33] Blom J.W., Vanderschoot J.P., Oostindier M.J., Osanto S., van der Meer F.J., Rosendaal F.R. (2006). Incidence of venous thrombosis in a large cohort of 66,329 cancer patients: Results of a recordlinkage study. J. Thromb. Haemost..

[34] Tao D.L., Bien J.Y., DeLoughery T.G., Shatzel J.J. (2017). Extended thromboprophylaxis with direct oral anticoagulants for medical patients: A systematic review and meta-analysis. Blood.

[35] Mulder F.I., Candeloro M., Kamphuisen P.W., Di Nisio M., Bossuyt P.M., Guman N., Smit K., Büller H.R., van Es N., CAT-Prediction Collaborators (2019). The Khorana score for prediction of venous thromboembolism in cancer patients: A systematic review and meta-analysis. Haematologica.

[36] Ay C., Dunkler D., Marosi C., Chiriac A.L., Vormittag R., Simanek R., Quehenberger P., Zielinski C., Pabinger I. (2010). Prediction of venous thromboembolism in cancer patients. Blood.

[37] Carrier M., Abou-Nassar K., Mallick R., Tagalakis V., Shivakumar S., Schattner A., Kuruvilla P., Hill D., Spadafora S., Marquis K. (2019). Apixaban to Prevent Venous Thromboembolism in Patients with Cancer. N. Engl. J. Med..

[38] Khorana A.A., Soff G.A., Kakkar A.K., Vadhan-Raj S., Riess H., Wun T., Streiff M.B., Garcia D.A., Liebman H.A., Belani C.P. (2019). Rivaroxaban for Thromboprophylaxis in High-Risk Ambulatory Patients with Cancer. N. Engl. J. Med..

[39] Agnelli G. (2019). Direct Oral Anticoagulants for Thromboprophylaxis in Ambulatory Patients with Cancer. N. Engl. J. Med..

[40] Mosarla R.C., Vaduganathan M., Qamar A., Moslehi J., Piazza G., Giugliano R.P. (2019). Anticoagulation Strategies in Patients With Cancer: JACC Review Topic of the Week. J. Am. Coll. Cardiol..

[41] Steffel J., Verhamme P., Potpara T.S., Albaladejo P., Antz M., Desteghe L., Haeusler K.G., Oldgren J., Reinecke H., Roldan-Schilling V. (2018). The 2018 European Heart Rhythm Association Practical Guide on the use of non-vitamin K antagonist oral anticoagulants in patients with atrial fibrillation. Eur. Heart J..

[42] Samuelson Bannow B.R., Lee A.Y.Y., Khorana A.A., Zwicker J.I., Noble S., Ay C., Carrier M. (2018). Management of anticoagulation for cancer-associated thrombosis in patients with thrombocytopenia: A systematic review. Res. Pract. Thromb. Haemost..

[43] Lee A.Y., Levine M.N., Baker R.I., Bowden C., Kakkar A.K., Prins M., Rickles F.R., Julian J.A., Haley S., Kovacs M.J. (2003). Randomized Comparison of Low-Molecular-Weight Heparin versus Oral Anticoagulant Therapy for the Prevention of Recurrent Venous Thromboembolism in Patients with Cancer (CLOT) Investigators. Low-molecular-weight heparin versus a coumarin for the prevention of recurrent venous thromboembolism in patients with cancer. N. Engl. J. Med..

[44] Lee A.Y., Bauersachs R., Janas M.S., Jarner M.F., Kamphuisen P.W., Meyer G., Khorana A.A., CATCH Investigators (2013). CATCH: A randomised clinical trial comparing long-term tinzaparin versus warfarin for treatment of acute venous thromboembolism in cancer patients. BMC Cancer.

[45] Raskob G.E., van Es N., Verhamme P., Carrier M., Di Nisio M., Garcia D., Grosso M.A., Kakkar A.K., Kovacs M.J., Mercuri M.F. (2018). Edoxaban for the Treatment of Cancer-Associated Venous Thromboembolism. N. Engl. J. Med..

[46] Young A.M., Marshall A., Thirlwall J., Chapman O., Lokare A., Hill C., Hale D., Dunn J.A., Lyman G.H., Hutchinson C. (2018). Comparison of an Oral Factor Xa Inhibitor With Low Molecular Weight Heparin in Patients With Cancer With Venous Thromboembolism: Results of a Randomized Trial (SELECT-D). J. Clin. Oncol..

[47] McBane R.D., Wysokinski W.E., Le-Rademacher J.G., Zemla T., Ashrani A., Tafur A., Perepu U., Anderson D., Gundabolu K., Kuzma C. (2020). Apixaban and dalteparin in active malignancy-associated venous thromboembolism: The ADAM VTE trial. J. Thromb. Haemost..

[48] Agnelli G., Becattini C., Meyer G., Muñoz A., Huisman M.V., Connors J.M., Cohen A., Bauersachs R., Brenner B., Torbicki A. (2020). Apixaban for the Treatment of Venous Thromboembolism Associated with Cancer. N. Engl. J. Med..

[49] Planquette B., Bertoletti L., Charles-Nelson A., Laporte S., Grange C., Mahé I., Pernod G., Elias A., Couturaud F., Falvo N. (2021). Rivaroxaban vs Dalteparin in Cancer-Associated Thromboembolism: A Randomized Trial. Chest.

[50] Schrag D., Uno H., Pam R., Rosovsky G., Rutherford C., Sanfilippo K.M., Villano J.L., Drescher M.R., Jayaram N.H., Holmes C.E. (2021). The comparative effectiveness of direct oral anti-coagulants and low molecular weight heparins for prevention of recurrent venous thromboembolism in cancer: The CANVAS pragmatic randomized trial. J. Clin. Oncol..

[51] Agnelli G., Buller H.R., Cohen A., Gallus A.S., Lee T.C., Pak R., Raskob G.E., Weitz J.I., Yamabe T. (2015). Oral apixaban for the treatment of venous thromboembolism in cancer patients: Results from the AMPLIFY trial. J. Thromb. Haemost..

[52] Riess H., Sinn M., Lohneis A., Hellmann M., Striefler J., Südhoff T., Pelzer U., Stahl M., Schlenska-Lange A., Krziwanie A. (2021). Improved Patient-reported Treatment Satisfaction with Rivaroxaban as Compared to Low Molecular Weight Heparins for Cancer Patients with Acute Venous Thromboembolism—Results from the CONKO-011 Trial [abstract]. Res. Pract. Thromb. Haemost..

[53] Cohen A.T., Maraveyas A., Beyer-Westendorf J., Lee A.Y.Y., Folkerts K., Abdelgawwad K., De Sanctis Y., Fatoba S., Bamber L., Bach M. (2021). Patient-reported outcomes associated with changing to rivaroxaban for the treatment of cancer-associated venous thromboembolism—The COSIMO study. Thromb. Res..

[54] Streiff M.B., Holmstrom B., Angelini D., Ashrani A., Bockenstedt P.L., Chesney C., Fanikos J., Fenninger R.B., Fogerty A.E., Gao S. (2018). NCCN Guidelines Insights: Cancer-Associated Venous Thromboembolic Disease, Version 2.2018. J. Natl. Compr. Cancer Netw..

[55] Key N.S., Khorana A.A., Kuderer N.M., Bohlke K., Lee A.Y.Y., Arcelus J.I., Wong S.L., Balaban E.P., Flowers C.R., Francis C.W. (2020). Venous Thromboembolism Prophylaxis and Treatment in Patients With Cancer: ASCO Clinical Practice Guideline Update. J. Clin. Oncol..

[56] Wojtukiewicz M.Z., Skalij P., Tokajuk P., Politynska B., Wojtukiewicz A.M., Tucker S.C., Honn K.V. (2020). Direct Oral Anticoagulants in Cancer Patients. Time for a Change in Paradigm. Cancers.

[57] Renner E., Barnes G.D. (2020). Antithrombotic Management of Venous Thromboembolism: JACC Focus Seminar. J. Am. Coll. Cardiol..

[58] Sørensen H.T., Mellemkjaer L., Olsen J.H., Baron J.A. (2000). Prognosis of cancers associated with venous thromboembolism. N. Engl. J. Med..

[59] Khorana A.A., Francis C.W., Culakova E., Kuderer N.M., Lyman G.H. (2007). Thromboembolism is a leading cause of death in cancer patients receiving outpatient chemotherapy. J. Thromb. Haemost..

[60] Kuderer N.M., Choueiri T.K., Shah D.P., Shyr Y., Rubinstein S.M., Rivera D.R., Shete S., Hsu C.Y., Desai A., de Lima Lopes G. (2020). Clinical impact of COVID-19 on patients with cancer (CCC19): A cohort study. Lancet.

